# Supplementary material for: HIVprotI: an integrated web based platform for prediction and design of HIV proteins inhibitors
Source: J Cheminform. 2018 Mar 9;10:12. doi: 10.1186/s13321-018-0266-y (PMC5845081; doi:10.1186/s13321-018-0266-y)
Supplement: Supplementary file 2 — Additional file 2. Source code of HIVProtI web server. [file 13321_2018_266_MOESM2_ESM.zip › HIVprotI_Source-code/hivprotI/weka-3-6-6/doc/weka/classifiers/trees/m5/package-summary.html]

weka.classifiers.trees.m5


---


|  |  |  |
| --- | --- | --- |
| **Overview** \| **Package** \| Class \| **Tree** \| **Deprecated** \| **Index** \| **Help** \| **Weka's home** | |  |
| **PREV PACKAGE**   **NEXT PACKAGE** | **FRAMES**    **NO FRAMES**     **All Classes** |  |


---

## Package weka.classifiers.trees.m5

| **Interface Summary** | |
| --- | --- |
| **SplitEvaluate** | Interface for objects that determine a split point on an attribute |

| **Class Summary** | |
| --- | --- |
| **CorrelationSplitInfo** | Finds split points using correlation. |
| **Impurity** | Class for handling the impurity values when spliting the instances |
| **M5Base** | M5Base. |
| **PreConstructedLinearModel** | This class encapsulates a linear regression function. |
| **Rule** | Generates a single m5 tree or rule |
| **RuleNode** | Constructs a node for use in an m5 tree or rule |
| **Values** | Stores some statistics. |
| **YongSplitInfo** | Stores split information. |

---


|  |  |  |
| --- | --- | --- |
| **Overview** \| **Package** \| Class \| **Tree** \| **Deprecated** \| **Index** \| **Help** \| **Weka's home** | |  |
| **PREV PACKAGE**   **NEXT PACKAGE** | **FRAMES**    **NO FRAMES**     **All Classes** |  |


---
